# Cognitive phenotyping of post-infectious SARS-CoV-2 patients

**DOI:** 10.1007/s10072-022-06130-8

**Published:** 2022-05-23

**Authors:** Edoardo Nicolò Aiello, Alice Radici, Gabriele Mora, Debora Pain

**Affiliations:** 1 PhD Program in Neuroscience, School of Medicine and Surgery, University of Milano-Bicocca, Monza, Italy; 2 IRCCS Istituti Clinici Scientifici Maugeri Di Milano, 20138 Milan, Italy

**Keywords:** SARS-CoV-2, COVID-19, Neuropsychology, Cognitive impairment: executive functioning

## Abstract

**Background:**

SARS-CoV-2 infection entails neuroinvasive, neuroinflammatory, and treatment-related features accounting for cognitive deficits in COVID-19-recovered patients. Although screening for such dysfunctions in this population is considered clinically relevant, contributions to cognitive phenotyping including premorbid and disease-related confounders are scarcely represented. This study thus aimed at describing the cognitive outcome at the function-/domain-level of post-infectious SARS-CoV-2 patients being already at risk (RCD +) or not (RCD −) for cognitive decline.

**Methods:**

Fifty-four COVID-19-recovered individuals were classified as either RCD + or RCD − according to medical records. The Mini-Mental State Examination (MMSE), Addebrooke Cognitive Examination-Revised (ACE-R), Frontal Assessment Battery (FAB), and Attentive Matrices (AM) were administered (*N* = 54, 34, 28, and 28 patients, respectively).

**Results:**

Prevalence of defective (cutoff = 24.89) MMSE scores was 24.3% in RCD + patients and 5.9% in the RCD − group. ACE-R-total below cutoff scores were less frequent (RCD + : 5.4%; RCD − : 5.9%). Abnormal performances at the FAB an AM were respectively detected in 18.9% and 8.1% of RCD + patients and 0% and 11.8% of the RCD − group. Within the ACE-R subtests, those assessing orientation, attention, and fluency were the most frequently impaired in both groups. Disease-related variables were mostly unassociated with cognitive measures.

**Discussion:**

Both RCD + and RCD − COVID-19-recovered individuals might show cognitive deficits within the dysexecutive-inattentive and amnesic spectrum. Non-instrumental, executive/attentive dysfunctions are predominant in this population and can be detected by both screening and domain-specific psychometric tests—although the latter might be more sensitive in RCD − patients.

## Introduction

SARS-CoV-2 infection entails neuroinvasive properties and systemic neuroinflammation that likely account for cognitive deficits occurring in COVID-19-recovered patients [1, 2]. Features related to COVID-19 management and treatment—e.g., ICU admission and steroidal therapy—were also shown to contribute to such cognitive sequelae [3–7].

Screening for cognitive dysfunction in this population has been suggested as clinically relevant regardless of the occurrence of neurological signs/symptoms [8], as it detrimentally affects patients’ prognosis and rehabilitative outcome [9, 10]. Hence, cognitive phenotyping of post-infectious SARS-CoV-2 patients is highly recommended.

Executive/attentive and episodic-memory dysfunctions have been highlighted as predominant features of the cognitive profile of post-COVID-19 patients [11–13]. However, comprehensive, domain-specific cognitive investigations also accounting for premorbid and disease-related confounders are still scarcely represented in this population [14–16]. However, it is mandatory to control for such intervening variables in order to provide valid data on the cognitive profile of COVID-19-recovered individuals. It is indeed still a matter of debate whether the cognitive toll of COVID-19 actually reflects the neurological effects of SARS-CoV-2 infection or is confounded by predisposing risk factors [17].

Thereupon, this study aimed at describing the cognitive outcome of post-infectious SARS-CoV-2 patients at the function/domain level by separately addressing those being already at risk (RCD +) or not (RCD −) for cognitive decline.

## Methods

### Participants

Data from *N* = 54 post*-*infectious SARS-CoV-2 patients referred to IRCCS Istituti Clinici Scientifici Maugeri of Milan (Northern Italy) between 2020 and 2021 were retrospectively collected (Table 1).

**Table 1:** Patients’ background and clinical features

Outcome		RCD +	RCD −	*p*†
*N*		37	17	-
Age (years)		70.30 ± 13.6 (36–93)	69.59 ± 12.06 (53–92)	.675
Sex (male/female)		15/22	10/7	.25
Education (years)		9.73 ± 4.2 (5–19)	9.59 ± 3.95 (5–18)	.985
Disease duration (days)		48.03 ± 23.35 (13–99)	53.47 ± 26.55 (16–94)	.595
Time from onset (days)		127.65 ± 114.11 (21–422)	77.59 ± 33.84 (22–154)	.226
Neurological conditions				
	Vascular	48.6%	-	
	Degenerative	8.1%	-	
	Neoplastic	5.4%	-	
	Epileptic	2.7%	-	
	Viral	5.4%	-	
	Cognitive	13.5%	-	
	Psychiatric	8.1%	-	
Disease severity	.223			
	Asymptomatic	16.2%	5.9%	-
	Mildly symptomatic	16.2%	17.6%	-
	Mild-to-moderate	37.8%	29.4%	-
	Moderate-to-severe	29.7%	47.1%	-
ICU		29.7%	47.1%	.175
Steroids		43.2%	47.1%	.1
Infection		24.3%	35.3%	.516

RCD +  = patients at risk for cognitive deficits; RCD −  = patients not at risk for cognitive deficits; *MMSE*, Mini-Mental State Examination; *MoCA*, Montreal Cognitive Assessment; *ICU*, intensive care unit. †*p*-values refer to either *χ*^2^ (categorical measures) or Mann–Whitney *U*/Kruskal–Wallis *H* (continuous measures). *Significant at *α* = .05

Similarly to Aiello et al. [14, 18], patients were sub-divided into either RCD + or RCD − . For a patient to be classified as RCD + , at least one neurological/psychiatric condition possibly affecting cognition had to be retrievable from either remote, recent, or COVID-19-related medical records. Clinical conditions presented by RCD + patients are described in Table 2 separately for remote, recent, and COVID-19-related medical history. By contrast, RCD − patients did not present with neurological/psychiatric risk factors for cognitive decline. The RCD + /RCD − classification was performed, based on the available medical record, by two independent authors (E. N. A. and D. P.) blinded to each other’s decision. Disagreements were solved through discussion and with the help of a third independent author (A. R.).

**Table 2:** Clinical presentations of RCD + patients

	Medical history		
	Remote	Recent	COVID-19 related
Vascular (***N***)			
Small vessel disease	7	-	1
Ischemic stroke	4	3	7
Hemorrhagic stroke	1	2	-
Brain aneurysm	1	-	-
Degenerative (***N***)			
Parkinson’s disease	2	-	-
Frontotemporal dementia	-	1	-
Neoplastic (***N***)			
Supratentorial brain cancer	1	1	-
Epileptic (***N***)			
Epilepsy	1	-	-
Viral (***N***)			
Posterior reversible encephalopathy syndrome	-	-	1
Post-infectious encephalomyelitis	-	-	1
Cognitive (***N***)			
Mild cognitive impairment	2	1	1
Intellectual disability	1	-	-
Psychiatric (***N***)			
Substance use disorder	2	-	-
Depression/anxiety	1	-	-
Delirium	-	-	2

Based on current guidelines [19], COVID-19 severity was graded as “asymptomatic”; “mildly symptomatic”; “mild-to-moderate” (requiring O_2_ therapy but not ventilation); and “moderate-to-severe” (requiring either non-invasive ventilation or ICU admission).

This study received approval by the local Ethics Committee (I.D.: 2494, 12 January 2021) and was conducted in accordance with the Declaration of Helsinki.

### Materials

Screening measures of cognitive efficiency—Mini-Mental State Examination (MMSE) [20, 21]; Addenbrooke’s Cognitive Examination—Revised (ACE-R) [20, 21]—and executive functioning—Frontal Assessment Battery (FAB) [22, 23]—were retrieved. The ACE-R encompasses subtests assessing Orientation and Attention (ACE-R-OA), Memory (ACE-R-M), Fluency (ACE-R-F), and Visuo-spatial abilities (ACE-R-VS) [23]. According to Aiello et al. [24], the FAB assesses verbal- (FAB-1) and motor-mediated (FAB-2) executive functioning, as well as inhibition (FAB-3). A domain-specific measure of attention was available for *N* = 28 patients—attentional matrices (AM) [25].

### Statistical analyses

SPSS 27 (IBM Corp., 2020) was used to analyze data.

In order to draw clinical judgments, MMSE, ACE-R, FAB, and AM scores were standardized according to the equivalent score (ES) method [26]. The ES method entails (1) adjusting raw scores for anagraphic-demographic predictors via linear models and then (2) converting adjusted scores into a 5-level ordinal scale (ES = 0, defective; ES = 1, borderline; ES = 2, “low-end” normal; ES = 3/4, normal).

Due to data distributions being often skewed and overdispersed, associations/predictions of interest were explored through non-parametric tests. When running analyses separately for RCD + and RCD − patients, *α* levels were Bonferroni-corrected (*α*_adjusted_ = 0.025). Missing data due to imputation issues were excluded pairwise.

## Results

Table 3 summarizes cognitive performances of RCD + and RCD − patients. MMSE scores were available for all patients, whereas ACE-R ones are for 34, and FAB and AM ones are for 28 ones. More specifically, ACE-R scores were available for 11 RCD − and 23 RCD + patients, FAB scores for 10 RCD − and 18 RCD + patients, whereas AM scores for 11 RCD − and 17 RCD + patients.

**Table 3:** Patients’ psychometric measures

Measure		RCD +		RCD −	
		Adjusted scores	Below-cutoff (%)	Adjusted scores	Below-cutoff (%)
MMSE (***N*** = 54)		27.50 ± 2.93 (20.42–30)	24.3%	27.82 ± 2.34 (21.18–30)	5.9%
ACE-R (***N*** = 34)					
	Total	87.38 ± 10.28 (59.59–100)	5.4%	90.93 ± 9.04 (69.4–100)	5.9%
	Attention and Orientation	16.69 ± 1.82 (11.86–18)	18.9%	17.04 ± 1.56 (12.1–18)	5.9%
	Memory	21.21 ± 4.48 (11.56–26)	5.4%	22.58 ± 3.9 (14.15–26)	5.9%
	Language	25.05 ± 2.2 (16.6–26)	2.7%	24.94 ± 1.93 (20.78–26)	0%
	Fluency	8.73 ± 2.11 (4.54–11.88)	8.1%	9.40 ± 2.4 (5.81–13.03)	5.9%
	Visuo-spatial abilities	13.66 ± 2.17 (7.27–17.27)	5.4%	14.53 ± 1.82 (11.15–16)	0%
FAB (***N*** = 28)	Total	13.97 ± 3.92 (5.79–18)	18.9%	15.45 ± 1.84 (13.06–18)	0%
	FAB-1	4.19 ± 1.46 (.61–5.86)	13.5%	4.64 ± 0.77 (3.10–5.98)	5.9%
	FAB-2	4.47 ± 1.83 (.76–6)	10.8%	5.62 ± .69 (3.72–6)	0%
	FAB-3	4.95 ± 1.23 (2.79–6)	2.7%	5.02 ± 1.14 (2.84–6)	0%
AM (***N*** = 28)		39.06 ± 9.23 (20.5–50.5)	8.1%	39.04 ± 11.06 (20–59.75)	11.8%

RCD +  = patients at risk for cognitive deficits; RCD −  = patients not at risk for cognitive deficits; *MMSE*, Mini-Mental State Examination; *ACE-R*, Addenbrooke’s Cognitive Examination-Revised; *FAB*, Frontal Assessment Battery; *FAB-1*, first two items of the FAB; *FAB-2*, second two items of the FAB; *FAB-3*, last two items of the FAB

RCD + (*N* = 37) and RCD − (*N* = 17) patients were balanced for all background and clinical variables (Table 1) and did not differ as to adjusted cognitive measures (49 ≤ *U* ≤ 309.5; *p* ≥ 0.07).

Prevalence of defective MMSE scores (*N* = 54) was 24.3% in RCD + patients and 5.9% in the RCD − group. ACE-R-total below-cutoff scores (*N* = 34) were less frequent (RCD + : 5.4%; RCD − : 5.9%). With respect to the FAB an AM, impaired performances were respectively detected in 18.9% and 8.1% of RCD + patients and 0% and 11.8% of the RCD − group. Within the ACE-R subtests, the ACE-R-AO and ACE-R-F were found to be the most frequently impaired in both RCD + (ACE-R-AO: 18.9%; ACE-R-F: 8.1%) and RCD − (ACE-R-AO: 5–9%; ACE-R-F: 5.9%) patients. The highest prevalence of defective performance among FAB subtests was found for the FAB-1 in both groups (RCD + : 13.5%; RCD − : 5.9%).

In both groups, no effects of disease severity (0 ≤ *H*(3) ≤ 6.49; *p* ≥ 0.078), ICU admission (1 ≤ *U* ≤ 124; *p* ≥ 0.078), steroidal treatment (8 ≤ *U* ≤ 123; *p* ≥ 0.093), and co-occurring infection (0 ≤ *U* ≤ 88; *p* ≥ 0.103) were detected on adjusted cognitive scores—with the exception of co-occurring infections on ACE-R-F (*z* =  − 2.65; *p* = 0.006) and ICU admission rates on FAB-3 scores (*z* =  − 2.35; *p* =  − 0.019) in RCD − patients. More specifically, ICU-admitted RCD − patients reported higher FAB-3 scores (*Mdn* = 5.96) vs. non-ICU-admitted ones (*Mdn* = 4.05), whereas RCD − patients suffering from co-occurring infections featured higher scores on the ACE-R-F (*Mdn* = 11.9) when compared to those who did not (*Mdn* = 7.77).

Disease duration and time from onset were mostly unassociated with cognitive measures in both groups (*r*_*s*_ ≤|.65|; *p* ≤ 0.3), except for disease duration being inversely related to ACE-R-L scores in RCD − patients (*r*_*s*_(11) =  − 0.71; *p* = 0.015) and positively with ACER-R total (*r*_*s*_(11) = 0.61; *p* = 0.002) and ACE-R-F (*r*_*s*_(11) = 0.51; *p* = 0.013) scores in the RCD + group.

## Discussion

The present study reports data on the cognitive profile of post-infectious SARS-CoV-2 patients by separately addressing those already at risk for cognitive decline (RCD +) and those who did not (RCD -). According to previous findings [5, 14], in both RCD + and RCD − patients, deficits were detected in both global cognitive efficiency (as assessed by MMSE and ACE-R-total scores) and specific function/domains (i.e., executive functioning/attention and memory, as assessed by the FAB, AM, and ACE-R-F and ACE-R-M scores).

The present results are also consistent with the notion of non-instrumental, dysexecutive-inattentive dysfunctions being predominant in this population [11, 13, 27, 28]. In this respect, it is worth noting that fluency tasks (as both comprised within the FAB-1 and the ACE-R-F subtests) proved to be effective in detecting dysexecutive features—especially in RCD − patients, who, by contrast, did not show FAB-2 and FAB-3 below-cutoff scores.

Attention deficits—as revealed by both I- (ACE-R-AO) and II-level measures (AM)—were detected in both groups [13]. Interestingly, the prevalence of below-cutoff AM scores was slightly higher in RCD − patients—suggesting that domain-specific tests might be able to detect impairment of attentive processes even in COVID-19-recovered individuals not already at risk for cognitive decline.

Notably, besides the ACE-R-F subtest, the only other ACE-R subtest yielding defective performances in RCD − patients was related to memory functions (ACE-R-M). In this regard, both subjective memory difficulties and objective memory deficits are among the most frequently reported cognitive symptoms by COVID-19-recovered individuals [15], which also yield in psychometric testing [29].

The present study also provides practitioners with evidence about the effectiveness of the ACE-R, FAB, and AM in detecting cognitive dysfunctions in post*-*infectious SARS-CoV-2 patients.

As to RCD + patients, the descriptive finding of an overall greater prevalence of defective cognitive performances when compared to the RCD − group was expected, as the former showed neurological/psychiatric conditions which might have already impacted on their cognitive functioning. However, it is noteworthy that, when addressing adjusted scores instead of prevalence values, no differences were detected in cognitive measures between these two groups. Although this finding should be regarded with extreme caution due to the small sample sizes, it is reasonable to postulate that, within the present RCD + cohort, patients did not present, at least at the group level, with cognitive dysfunctions of such a severity that would have allowed for them to be discriminated form RCD − ones.

Finally, poorly interpretable findings raised on the interplay between cognition and disease-related features in COVID-19-recovered individuals (i.e., higher ACE-R-F scores in RCD − patients suffering from co-occurring infections and higher ACE-R-total and ACE-R-F scores in RCD + patients with longer disease). However, the present data pointing out at better executive outcomes (FAB-3) in ICU-admitted patients and at a greater language involvement (ACE-R-L) in patients with higher disease duration, consistently with previous contributions [1, 16, 30]. Such findings might respectively suggest that (i) ICU-admitted patients may suffer less than expected from hypoxic aftermaths of pneumonia to the brain, notwithstanding the “aggressive” treatment and (ii) a longer disease duration may negatively influence cognitive functions due to prolonged neuroinvasive and neuroinflammatory processes. Nonetheless, it must be noted that contrasting evidence has recently emerged as for ICU admission, namely that admitted patients may have worse cognitive outcomes [7].

This study does present with several limitations. First, the sample size is relatively small and data were retrieved from a single clinic. Moreover, since this was a retrospective investigation, not all cognitive measures herewith addressed were available for all patients. Both these elements may to an extent limit the external validity of the present findings. Furthermore, a restricted range of cognitive tools (mostly screening ones) were addressed within this study: further investigations are thus desirable that take into account a thorough cognitive battery.

In conclusion, the present work shows that COVID-19-recovered individuals regardless of the pre-existence of risk for the cognitive decline might show cognitive deficits within the dysexecutive-inattentive and amnesic spectrum. Non-instrumental, executive/attentive dysfunctions are predominant in this population and can be detected by both I- and II-level psychometric tests—although the latter might be more sensitive to such impairments and should thus be preferred in patients who are not already at risk for cognitive decline.
